# Meetings of Societies

**Published:** 1947

**Authors:** 


### Meetings of Societies

### Bristol Medico-Chirurgical Society

PROGRAMME FOR THE 1947–48 SESSION

December 10th.—Clinical Meeting at the Royal Fort.

January.—Dr. Campbell: "**Nervous Diseases in Animal and Man.**"

February.—Dr. Thompson, of Belfast, to read "**Malignant Disease of the Lung.**"

